# **The Impact of Hepatitis B Infection on Outcome of Kidney Transplantation: A Long-Term Study**

**Published:** 2010-05-01

**Authors:** B. Einollahi, S. M. Alavian, M. Lessan-Pezeshki, N. Simforoosh, M. H. Nourbala, Z. Rostami, V. Pourfarziani, E. Nemati, M. Sharafi, M. Nafar, F. Pour-Reza Gholi, A. Firoozan

**Affiliations:** 1 *Nephrology and Urology Research Center, Baqiyatallah University of Medical Sciences, Tehran, IR Iran*; 2 *Baqiyatallah Research Center for Gastroenterology and Liver Disease, Baqiyatallah University of Medical Sciences, Tehran, IR Iran*; 3 *Department of Nephrology, Tehran University of Medical Sciences, Tehran, IR Iran*; 4 *Renal Transplantation Center, Shahid Beheshti University of Medical Sciences, Tehran, IR Iran*

**Keywords:** Kidney transplantation, Hepatitis B virus, Hepatitis C virus

## Abstract

Background: With the success of kidney transplantation, liver disease has emerged as an important cause of morbidity and mortality in kidney recipients.

Objective: To determine the impact of hepatitis B virus (HBV) infection on patients and graft survival in both short- and long-terms.

Methods: 99 renal transplant patients infected with HBV on follow-up in two major transplant centers were included in a retrospective study. These patients were grafted between 1986 and 2005 and divided into two groups: (1) those only positive for hepatitis B surface antigen (HBsAg) and (2) those who were also positive for hepatitis C virus antibodies (HCV Ab).

Results: There were 88 patients with HBsAg^+^ and 11 with both HBsAg^+^ and HCV Ab^+^. The mean±SD age of patients was 38.8±13.2 years, and the median follow-up after transplantation was 19 months. Although not significant, the allograft survival rate in the first group (HBV^+^) was better compared to that in the second group (HBV^+^ and HCV^+^); 1, 5 and 10 years graft survival rates were 91, 77 and 62 in the first group and 70, 56 and 28 in the second group, respectively (P=0.07). The overall mortality was 5% (4 of 88) in the first and 27% (3 of 11) in the second group (P=0.02).

Conclusion: Renal allograft recipients with HBV and HCV infections has a poor survival rate compared to patients with only HBV infection. However, there is no significant difference in terms of renal graft survival between the two groups.

## INTRODUCTION

Chronic hepatitis B virus (HBV) infection has remained a public health concern among infected renal allograft recipients because it results in an increased risk of morbidity and mortality due to liver failure [1]. Liver diseases are relatively frequent and important complications after renal transplantation. The prevalence of liver disease ranges from 9% to 34% of renal transplant recipients. Hepatitis B and C infections are the most important causes of chronic liver disease [2]. Kliem, *et al* [3] have recently shown that the prevalence of HBV was 2.9%, of HCV was 8.7% and of coinfection with HBV and HCV was 0.4%. However, there are geographical variations in prevalence of hepatitis following kidney transplantation [3]. A survey involving 12 transplant centers from 11 countries in Asian-Pacific region showed that 1%–25% of kidney transplant recipients were infected with HBV, and up to 60% of these subjects showed abnormal liver biochemistry [4]. Nevertheless, limited data are available about natural history of HBV infection in Iran. Therefore, we conducted this retrospective study to determine the impact of HBV infection on patients and graft survival in both short- and long-terms.

## MATERIALS AND METHODS

We studied renal transplant recipients with hepatitis B infection who were grafted between 1986 and 2005 in two leading transplant centers in Iran. These patients were divided into two groups: (1) those only positive for hepatitis B surface antigen (HBsAg), and (2) those who were also positive for hepatitis C virus antibodies (HCV Ab). The median post-transplantation follow-up period was 19 months. Since 1995, infected patients with hepatitis B were treated by lamivudine.

Data were analyzed by SPSS for Windows ver 15.0. Categorical variables were presented as relative frequencies and analyzed using χ^2 ^or Fisher’s exact test. Continuous variables were presented as mean±SD. The impact of HBV infection on overall patients’ and graft survival rates in both short- and long-terms were calculated using the Kaplan-Meier survival analysis; comparisons were made using log-rank analysis. A P value <0.05 was considered statistically significant.

## RESULTS

Ninety-nine renal transplant recipients infected with HBV; 88 were only HBsAg-positive and 11 had positive with both HBsAg and HCV Ab. The male:female ratio was 76:23. The mean±SD age of studied patients was 38.8±13.2 years. The allograft survival rate in the first group (HBV^+^) was higher than that in the second group (HBV^+^ and HCV^+^) though the difference was not statistically significant (P=0.07) (Table 1). The overall mortality was 5% (4 of 88) in the first group and 27% (3 of 11) in the second group (P=0.02). One patient with HBV developed squamous cell carcinoma of the skin and another developed Kaposi’s sarcoma.

**Table 1:** Graft survival rates in the studied groups

Graft Survival	Only HBsAg^+^	HBsAg^+^ and HCV Ab^+^
One year (%)	91	70
Five years (%)	77	56
Ten years (%)	62	28

## DISCUSSION

It is known that prolonged immunosuppressive therapy after kidney transplantation enhances viral replication and promotes liver injury [1]. We studied the impact of HBV infection on patients and graft survival rates in renal transplants. The association of a higher mortality in renal transplant patients with HBs antigenemia has been confirmed by several authors [5, 6]. In comparison, the mortality rate in our HBsAg^+^ recipients was lower than those reports [5, 6]. On the other hand, coinfection with HBV and HCV carried a poor prognosis when compared to patients infected only with HBV (mortality rate: 27% *vs* 4.5%). The combined effect of HBV and HCV shows a high risk for graft loss and mortality [5]. However, in the current study, the allograft survival rate in HBV^+^ patients was better than that in patients with HBV^+^ and HCV^+^. However, no significant difference was seen in allograft survival rates between the two groups.

Park, *et al*, showed relatively favorable outcomes in HBV^+^ renal transplant patients receiving lamivudine [7]. Lamivudine was well tolerated without significant side effects in our patients and it can be responsible for the favorable outcomes.

## CONCLUSION

Patient and graft survival in HBV-infected recipients is promising. Therefore, HBsAg^+^ patients should not categorically be rejected for renal transplantation unless they also have associated chronic active hepatitis. However, recipients with mixed HBV and HCV infections had a poor survival rate compared to those infected with only HBV. 

## CONFLICT OF INTEREST:

None declared.
